# Temporal Dynamics of Treatment Receipt in a Text Message Intervention for Physical Activity: Single-Group, Within-Person Trial

**DOI:** 10.2196/14270

**Published:** 2020-04-22

**Authors:** David E Conroy, Chih-Hsiang Yang, Stephanie T Lanza, Joshua M Smyth, Constantino M Lagoa

**Affiliations:** 1 Department of Kinesiology The Pennsylvania State University University Park, PA United States; 2 Department of Exercise Science University of South Carolina Columbia, SC United States; 3 The Edna Bennett Pierce Prevention Research Center Department of Biobehavioral Health The Pennsylvania State University University Park, PA United States; 4 Department of Biobehavioral Health The Pennsylvania State University University Park, PA United States; 5 Department of Electrical Engineering & Computer Science The Pennsylvania State University University Park, PA United States

**Keywords:** short message service, patient engagement, mHealth, physical activity, sedentary behavior

## Abstract

**Background:**

Mobile technology has increased the reach of health behavior interventions but raised new challenges in assessing the fidelity of treatment receipt. Fidelity can be compromised if participant fatigue or burden reduces engagement, leading to missed or delayed treatments for just-in-time interventions.

**Objective:**

This study aimed to investigate the temporal dynamics of text message receipt confirmations.

**Methods:**

Community-dwelling adults (N=10) were sent five text messages daily for 4 months (5598 messages sent in total), with a financial incentive to confirm receipt of 75% or more messages.

**Results:**

Overall, the message receipt confirmation rate was very high (5504/5598, 98.32%) and timely (eg, two-thirds of confirmations within 2 min). Confirmation times were slightly slower on weekends (vs weekdays) and as a function of the cumulative time in the study. Neither time of message delivery nor message content was associated with message confirmation latencies.

**Conclusions:**

Participants receiving financial incentives to confirm text message receipt exhibit extremely high and fast confirmation rates, although receipt confirmations were somewhat less timely on weekends (vs weekdays) and later in the intervention. The social calendar and treatment fatigue should be considered when planning text message–based interventions, especially if treatments are intended for a just-in-time delivery that requires extended engagement and precise timing.

## Introduction

### Background

Digital tools, such as mobile phones, have transformed the form and reach of many health behavior interventions. It is now possible to deliver intensive treatments to people in the natural context of their daily lives [1]. Mobile health (mHealth) interventions have shown considerable promise as being efficacious for modifying a number of preventive health behaviors [2]. Text messages (ie, SMS) have emerged as a popular mode of mHealth intervention delivery because over 90% of American adults own a cell phone and over 80% use their phones for text messaging [3,4]. SMS intervention effects have been quite variable across a range of health behaviors and, more specifically, have not produced consistent changes in physical activity [2,5,6]. One possible source of that variation involves the fidelity of treatment receipt: If people do not receive or read messages in a timely fashion, their full effects are unlikely to be realized, particularly for just-in-time interventions that are sensitive to synchronizing the timing of message receipt with moments of opportunity or vulnerability. Little is known about the temporal dynamics of treatment receipt in SMS interventions generally or for just-in-time messaging interventions specifically. This knowledge gap presents a barrier to optimally engaging patients in just-in-time messaging interventions. This study documented the rate of message confirmations within a variety of time windows and estimated associations between time-varying (within-person) factors and an important indicator of timely treatment receipt, namely, the latency of message receipt confirmations.

### Treatment Fidelity

The National Institutes of Health (NIH) Behavioral Change Consortium defined the scope of fidelity as spanning study design; staff training; and intervention delivery, receipt, and enactment [7,8]. High fidelity is essential for drawing causal inferences about intended intervention effects. One prominent threat to mHealth treatment fidelity is insufficient user engagement with the behavior change intervention or its techniques [9]. A recent study of mobile apps for mental health indicated that users’ daily rate of opening the apps dropped by 80% in the first 10 days after initially installing and opening the app [10]. This decline in engagement reduces mHealth treatment fidelity but is somewhat unavoidable when users are required to initiate their interactions with intervention content. One way to reduce that friction and boost engagement is to push intervention content to users using SMS messages or notifications. With just-in-time or ecological momentary intervention approaches, the intervention can be conceptualized as a series of individual intervention doses (eg, SMS messages) delivered over time [1,11]. Intervention doses that are not delivered, received, or enacted will introduce discrepancies between intended and enacted treatment doses and undermine treatment fidelity. If the individual intervention doses require precise timing to coincide with moments of opportunity or vulnerability, it is essential for those messages to be received when delivered [12]. Doses that are not received as intended are unlikely to change behavioral target actions or clinical outcomes [13]. Moreover, when mHealth interventions fail, a lack of treatment fidelity makes it difficult to understand whether that failure was because of failures in study design (eg, inert intervention content and insufficient dosing), failures in treatment delivery, failures in treatment receipt, or failures to enact prescribed actions. Yet granular data on treatment receipt from just-in-time interventions have not been widely reported to date, and little is known about the factors that influence those dynamics.

It can be challenging to verify whether participants read SMS messages as they are received (or at all). Simply verifying that a message was opened in a timely fashion would eliminate the need to assume that messages are always received as sent. Such data would help to rule out the possibility that messages were accumulating undelivered or that long delays existed between message transmission and receipt. If participants were asked to confirm message receipt with a response, those responses could signal that messages were at least delivered, opened, and inspected enough to determine whether a confirmation was needed (ie, discriminating between intervention-related messages and personal messages). Such message confirmations could indicate that some level of treatment was received (although they do not provide direct evidence that the message was understood or enacted). Thus, overall confirmation response rates and latencies can serve as approximate indicators of the fidelity of text message treatment receipt.

### Treatment Fidelity as a Dynamic Process

Several dynamic factors may influence the latency of text message receipt confirmations, including treatment fatigue and timing. Treatment fatigue describes “psychological fatigue associated with treatment engagement” [14]. The effort required to open and read a single text message is of course minimal, but this activity requires one to interrupt other ongoing activities and shift attention so cumulative effort may be substantial (ie, across multiple messages). Unless messages are highly rewarding (ie, interesting or enjoyable), intervention burden can accumulate to create fatigue and may reduce adherence to reading messages in a timely fashion; eg, self-reports of cessation fatigue during smokers’ quit attempts have been associated with reduced success [15]. We are not aware of any studies on behavioral indicators of treatment fatigue experienced during intensive SMS interventions for physical activity or for extended periods of time. This study examined changes in overall response confirmation rates across a long-term SMS intervention when participants are vulnerable to treatment fatigue and related processes that would undermine the fidelity of treatment receipt (eg, habituation).

In an intensive SMS intervention, the timing of message distribution may also vary on daily and hourly time scales. For example, differences in discretionary time availability because of work or personal obligations may produce weekday-weekend differences in the dynamics of treatment receipt. Similarly, the time of day when messages are sent may impact how quickly people can read and respond to confirm receipt. These hypotheses about differences in response confirmation times by day-of-week or time of message delivery were treated as exploratory.

### Objective

This study was designed to investigate the dynamics of treatment receipt in an intensive and long-term SMS intervention to promote physical activity. For 4 months, participants received five SMS messages daily, each randomly drawn from large pools of messages that focused on (1) social-cognitive processes associated with physical activity (*move more*, 101 messages), (2) social-cognitive processes associated with limiting sedentary behavior (*sit less*, 101 messages), or (3) non–health-related general facts (trivia, 254 messages). We hypothesized that the latency of SMS receipt confirmations would increase as a function of the number of days since the beginning of the intervention. We also hypothesized that receipt confirmation latency would vary as a function of the day of the week and the time of day when messages were delivered, but we made no hypothesis about the direction of these associations. Finally, we evaluated whether message content (*move more* and *sit less*) influenced message confirmations relative to the non–health-related general fact messages.

## Methods

### Participants and Procedures

Community advertisements were used to recruit participants for a study to evaluate a 16-week SMS intervention to promote physical activity. Participants (9/10, 90% female) ranged from 22 to 47 years in age with a mean of 34.3 (SD=8.99) years. This sample was mostly white (9/10, 90%) and not Hispanic or Latino (10/10, 100%) with full-time employment (8/10, 80%). Participants varied in their marital status (5/10, 50% single; 4/10, 40% married, and 1/10, 10% divorced) and status as parents (6/10, 60% had children). Maximum educational attainments ranged from a high school diploma to a doctoral degree, but most of the sample (60%) had not completed a Bachelor’s degree.

For the 16 weeks of the study, participants received five text messages daily. A static *Do Not Disturb* window was fixed for all participants between 8:00 PM and 8:00 AM. Messages were delivered using an online service on a semirandom schedule within equal-sized segments under the constraint that consecutive messages were separated by at least 60 min [16]. For all messages, participants were instructed to confirm receipt by replying to each message as quickly as possible after reading it. Responses could be as simple as a single letter to minimize burden. As an incentive, participants received a weekly payment (US $15/week) for responding to 75% of the text messages each week. Participants who responded to at least 75% of the messages within 2 min during a month entered a drawing for a US $100 bonus. Details about the study that are not relevant to this report (eg, activity monitoring) have been reported elsewhere [17].

### Measures

Response confirmation latencies were measured in seconds (to the second decimal place) from the time the intervention SMS was sent until the corresponding SMS confirmation was received. Confirmations received in less than 1 second (n=94; <1%) were deemed implausible and attributed to technical errors, and so they were recoded as missing values.

### Data Analysis

Response latencies were positively skewed. A natural log transformation was implemented to normalize the distribution of scores. Multilevel models were estimated to model the duration of confirmation latencies [18]. A dummy variable was created to represent weekend days (weekdays were set as the reference category). Time of day was coded as minutes since 3 AM because of the observed SMS inactivity in the early morning hours. A pair of dummy variables were created to represent message content related to increasing physical activity or decreasing sedentary behavior (general fact messages were set as the reference category).

## Results

A total of 5598 SMS messages were sent and 5504 confirmations (5504/5598, 98.3%) were received. Confirmation responses were received very quickly overall, with most within 33 seconds (2690/5504, 49.87%), 2 min (3676/5504. 66.79%), or 5 min (4108/5504, 74.64%; percentages are cumulative). The distribution of response times had a heavy tail that was normalized after implementing a natural logarithm transformation. The intraclass correlation for the transformed score was 0.08. Given the minimal proportion of confirmation time variance that existed between people and our limited sample size, the multilevel model regressed transformed confirmation latencies on the hypothesized within-person predictors.

As shown in Table 1, inferred treatment fatigue (as indexed by day in study) was associated with a small, but statistically significant, increase in the transformed latency of response confirmations. Saturdays and Sundays were also associated with significantly longer transformed response confirmation latencies than weekdays (the untransformed equivalent of approximately 3.8 seconds longer for weekend response confirmations), but these differences were relatively small in absolute terms. Neither message delivery time nor message content was associated with transformed response confirmation latencies.

**Table 1:** Multilevel regressions of message confirmation latencies.

Variable	Beta	SE	*P* value
Treatment fatigue (study day)	.003^a^	0.001	.003
Message delivery time of day	.00	0.00	.62
Weekends (vs weekdays)	.40^a^	0.10	<.001
Physical activity (vs general) content	−.007	0.03	.83
Sedentary behavior (vs general) content	.07	0.06	.26
Residual variance (within person)	3.09^a^	0.25	<.001
Mean (between person)	−.09	0.20	.67
Variance (between person)	.28^a^	0.09	.002

^a^*P*<.01.

## Discussion

The objective of this brief report was to examine temporal correlates of the inferred fidelity of treatment receipt in an intensive SMS intervention. Results indicated that (1) there was a very high and sustained rate of message confirmations, likely because of (in full or in part) the incentive program, and (2) the social calendar and treatment fatigue, but not message timing or content, were associated with small but reliable latency differences in participant confirmations via SMS.

### Principal Findings

The very high message confirmation rate (5504/5598, 98.3%) was impressive given the duration (4 months) and intensity (five times daily) of the SMS intervention. Treatment fatigue was evidenced by the small, but statistically significant, increased delays before message confirmations were received as cumulative exposure to the intervention increased. This finding provides evidence of a potential barrier to treatment fidelity in SMS-based interventions requiring high temporal precision, particularly those with lengthy or intensive SMS schedules that require extended engagement, although we note that overall response rates were outstanding and the delays were of very small magnitude in the majority of cases. It should be noted that it is also not clear whether the delays were due to the burden of mentally processing message content, the workflow for responding to individual messages, or other factors (eg, being out of a service area or in airplane mode). Regardless of the source, a lack of fidelity in terms of treatment receipt can reduce confidence in inferences about intended treatment effects, particularly if fidelity was not evaluated and accounted for when estimating effects [8]. Research on chronic disease management also has shown that other indicators of treatment fatigue adversely impact adherence [19,20]. The relatively weak treatment fatigue observed in this study raises questions about whether such fatigue could be sufficient to reduce treatment enactment or modify behavior change. This issue should be monitored when implementing future SMS interventions to provide an empirical basis for answering this question.

As expected, the social calendar was associated with differences in the fidelity of treatment receipt: message confirmations were, on average, 3.8 seconds slower on weekends than weekdays, possibly because of reduced availability. Prior work has defined availability as a state in which a person “is capable of engaging in an incoming, unplanned activity” (p. 913), and—based on multiple wearable sensors used to infer availability—demonstrated greater availability on weekends than weekdays [21]. Taken together, these findings suggest that participants may be available for—but slightly less motivated to receive—interventions on weekends than on weekdays. However, the magnitude of differences observed in this study (and in the context of a financial reward for compliance) does not appear to be sufficient to represent a substantial threat to the fidelity of intervention receipt. Thus, this conclusion may well be limited to approaches that incentivized responses and may differ substantially when participants are not incentivized. It also may not generalize to people with different social calendars (eg, shift workers and stay-at-home parents). Future research can benefit from mixed method approaches to identify reasons for differential availability or motivation that can explain these within-person associations.

Neither message delivery time nor message content was associated with the fidelity of treatment receipt. Incentivized SMS confirmation receipts may be resistant to self-regulatory fluctuations because of changes in desire or impaired affective and cognitive functioning as the day progresses [22]. This study compared three types of SMS messages across a fixed 12-hour period of the day. To strengthen conclusions about message properties, future work should investigate the dynamics of treatment receipt for a more diverse array of health behaviors and across a wider time interval.

### Limitations

This study provided insights into how the fidelity of SMS treatment receipt can vary over time but also had several limitations. First, the design was not experimental, so causal inferences cannot be drawn based on these findings. Second, the sample size was small and homogenous, so results may not generalize to more representative populations, populations with different motivational profiles, or, as mentioned earlier, individuals who have different social calendars. Owing to the limited sample size, a two-level model was estimated (repeated measures of message confirmations within person). Future work with larger samples should consider a three-level model to separate the day-in-study variance from the time-of-day variance. Third, the financial incentive to confirm message receipt and the overall SMS confirmation rate were high. These findings may not generalize to contexts without financial incentives (although it is worth noting that, even with these incentives, treatment receipt was still weakly associated with treatment fatigue and day of week). It is possible that different patterns exist when participants are not incentivized to confirm message receipt or if incentives have different contingencies (eg, targeting latency or frequency alone). It seems likely that overall confirmation rates would likely be lower under either of those conditions, but research is needed to test that hypothesis. Fourth, participants’ availability was not screened to determine whether they could receive messages (eg, Is the person sleeping? Is the person driving? Is the person exercising already?), but high response rates were obtained using static timeframes for message delivery. Finally, message content was drawn from a scripted message bank with limited repetition; it is possible that fatigue may be a greater threat in contexts with less novelty and greater message repetition.

### Conclusions

We conclude that sustained high treatment fidelity, even in contexts with high-density responding (5 times/day) for sustained durations (4 months), is possible—at least under circumstances where there are financial incentives for confirming SMS treatment receipt. Although overall rates were very high, we also found some evidence that treatment fidelity remains is a dynamic process. Depending on the pattern of message receipt, treatment may be slightly biased toward certain situations. Specifically, SMS-based treatment receipt was slightly faster on weekdays (compared with weekends), and slowly decayed as the intervention continued over a 4-month period. Failures to receive interventions as intended may be one factor that helps explain the lack of consistent effects of mHealth physical activity interventions and variation in SMS-based effects for other health behaviors [2,5], although we again note that overall response in this context was exceptionally high. As such, the dynamics of treatment receipt should be monitored because this process is not static. These preliminary findings can help to inform best practices for using SMS (or a similar technology such as smartphone notifications) to deliver just-in-time interventions that depend on people receiving interventions at specific moments of vulnerability or opportunity.

## Abbreviations

mHealth: mobile health
